# Umbilical endometriosis: a case series

**DOI:** 10.1186/s13256-020-02492-9

**Published:** 2020-09-07

**Authors:** Dorothy Makena, Timona Obura, Steve Mutiso, Felix Oindi

**Affiliations:** Department of Obstetrics and Gynecology, Aga Khan University Hospital Nairobi, P.O. Box, Nairobi, 30270-00100 Kenya

**Keywords:** Umbilical endometriosis, Umbilical swelling, Cyclical pain, Surgical excision

## Abstract

**Background:**

Endometriosis is the presence of endometrial tissue outside the uterine cavity. The lesions are typically found in the pelvic cavity but can occur in other extrapelvic areas. Umbilical endometriosis, also known as Villar’s node, is a rare disease comprising 0.5–1% of all extrapelvic disease. It commonly presents with cyclical pain and bleeding from an umbilical nodule.

**Case series:**

We present a retrospective case series of five African patients with umbilical endometriosis diagnosed and treated between July 2015 and February 2019 at a tertiary health facility. The patients were aged between 31 and 47 years, and all presented with an umbilical swelling and pain. They had lesions with diameters ranging from 1.6 cm to 4 cm. The duration of symptoms ranged between 3 and 60 months. Their diagnoses were made on the basis of clinical presentation followed by surgical excision. In all the cases, diagnosis was confirmed by histopathology with no malignancy detected.

**Conclusion:**

Umbilical endometriosis is a rare condition that should be considered as a differential diagnosis in women with umbilical lesions. Diagnosis is mostly clinical; most patients present with umbilical swelling, cyclical pain, and bleeding or discharge. Imaging has a limited role. Surgical excision is the treatment of choice with low risk of malignancy or recurrence.

## Background

Endometriosis is the presence of endometrial tissue outside the uterine cavity. It is a benign condition affecting 10–15% of women [1]. It classically affects women of reproductive age. The lesions occur mostly on pelvic sites involving the ovaries, uterosacral ligaments, ovarian fossa, cul-de-sac, and bladder in that order [2]. Extrapelvic endometriosis occurs less commonly. The extrapelvic sites include the diaphragm, pulmonary, urinary tract, gastrointestinal tract, brain, and cutaneous endometriosis. Umbilical endometriosis is rare, and it comprises 0.5–1% of all extrapelvic disease. Although rare, umbilical endometriosis is the commonest type of cutaneous endometriosis [3].

Umbilical endometriosis can be categorized as primary when it occurs spontaneously or secondary when it occurs following laparoscopic or open procedures, the latter being more common [4]. Primary umbilical endometriosis was first described by Villar in 1886; therefore, it is also known as Villar’s nodule [5]. The pathogenesis of endometriosis is not well understood. Postulated theories include Sampson’s theory of retrograde menstruation, which is the commonest, coelomic metaplasia, induction theory, embryonic Mullerian rests, bone marrow stem cell theory, and hematogenous/lymphatic spread. The theory favored in the case of umbilical endometriosis is hematogenous/lymphatic spread where there is coexisting pelvic endometriosis. Isolated umbilical endometriosis could develop from metaplasia of urachal remnants [6]. The aim of this study was to evaluate the clinical characteristics, presentation, diagnosis, and management of umbilical endometriosis in view of the literature.

## Case series

This is a retrospective, single-center, consecutive case series of African patients managed in a private tertiary health facility in Nairobi, Kenya. We reviewed five cases of patients with histology-confirmed umbilical endometriosis who were managed at Aga Khan University Hospital, Nairobi, between July 2015 and February 2019. The patients were reviewed in the gynecology clinic, where they presented with an umbilical swelling with cyclical pain and bleeding/discharge. A clinical evaluation based on history and physical examination was done, followed by surgical excision of the lesion. The specimen was taken for histological diagnosis, which confirmed umbilical endometriosis in all the cases. The diagnosis was made when there was identification of endometrial glands and stroma, areas of focal hemorrhage or chronic inflammation, and presence of macrophages with hemosiderin pigments.

We analyzed age, parity, duration of symptoms, chief presentation, associated symptoms, size of the lesion, management, and histopathological diagnosis of the five patients included (Table 1). All the patients authorized informed consent in the medical records at admission.

**Table 1:** Clinical features and treatment

Patient	Age (years)	Parity	Duration of symptoms (months)	Chief presentation	Associated symptoms	Size of lesion in largest dimension (cm)	Imaging	Management	Histology
1	33	0 + 0	8	Umbilical swelling, cyclic discharge	No dysmenorrhea, normal flow	1.6	MRI	Excision	Confirmed
2	46	6 + 0	24	Umbilical swelling, cyclical pain, and bleeding	–	4	–	Excision	Confirmed
3	47	1 + 0	60	Umbilical swelling, cyclical pain, and bleeding	Dysmenorrhea, heavy menses	3.5	US	Excision	Confirmed
4	31	0 + 0	12	Umbilical swelling, cyclical pain, and bleeding	Dysmenorrhea, normal flow	3	–	Excision	Confirmed
5	43	2 + 0	3	Umbilical swelling, cyclical pain, and bleeding	Severe dysmenorrhea, normal flow	3	–	Excision	Confirmed

*MRI* Magnetic resonance imaging, *US* Ultrasound

The clinical features and treatment details of the patients are summarized in Table 1. The mean age of the patients was 40 years, with a range from 31 to 46 years. Two of the patients were nulliparous, and the other three had previous deliveries ranging from one to six deliveries. None had a miscarriage. One of the patients had two previous cesarean deliveries, whereas the others had vaginal deliveries. No other previous abdominal surgeries were reported among the patients.

The mean duration of symptoms prior to presentation was 21.4 months, with a range from 3 to 60 months. The preoperative diagnosis was clinical in all the cases (100%). The presentation was similar, with all having an umbilical swelling (100%), cyclical pain and bleeding in four of five (80%), with one having umbilical discharge instead of bleeding. Three of the patients reported associated symptoms of severe dysmenorrhea (60%), with one having heavy menses, too. One patient was being seen in follow-up for subfertility with bilateral tubal blockage.

**Fig. 1:**
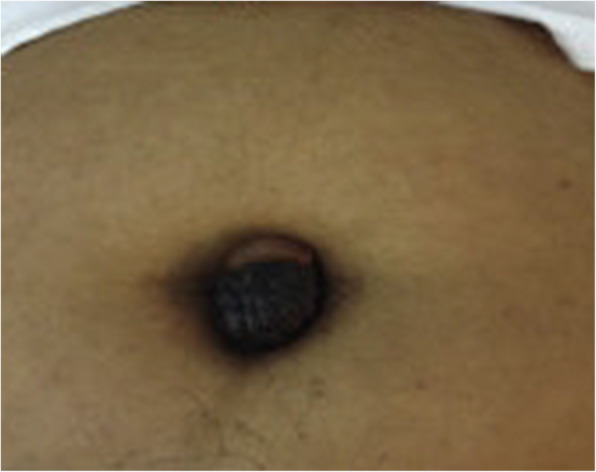
Hyperpigmented umbilical nodule

The largest dimension of the umbilical lesion (Fig. 1) ranged from 1.6 to 4 cm (mean 3.02 cm). One patient had preoperative imaging with magnetic resonance imaging (MRI), which revealed a 1.6-cm umbilical lesion suggestive of umbilical endometriosis with normal pelvic findings. Another patient had a preoperative pelvic ultrasound that revealed multiple intramural fibroids, the largest about 3 cm, with a right ovarian simple cyst about 3 cm. The other three patients did not have any imaging.

**Fig. 2:**
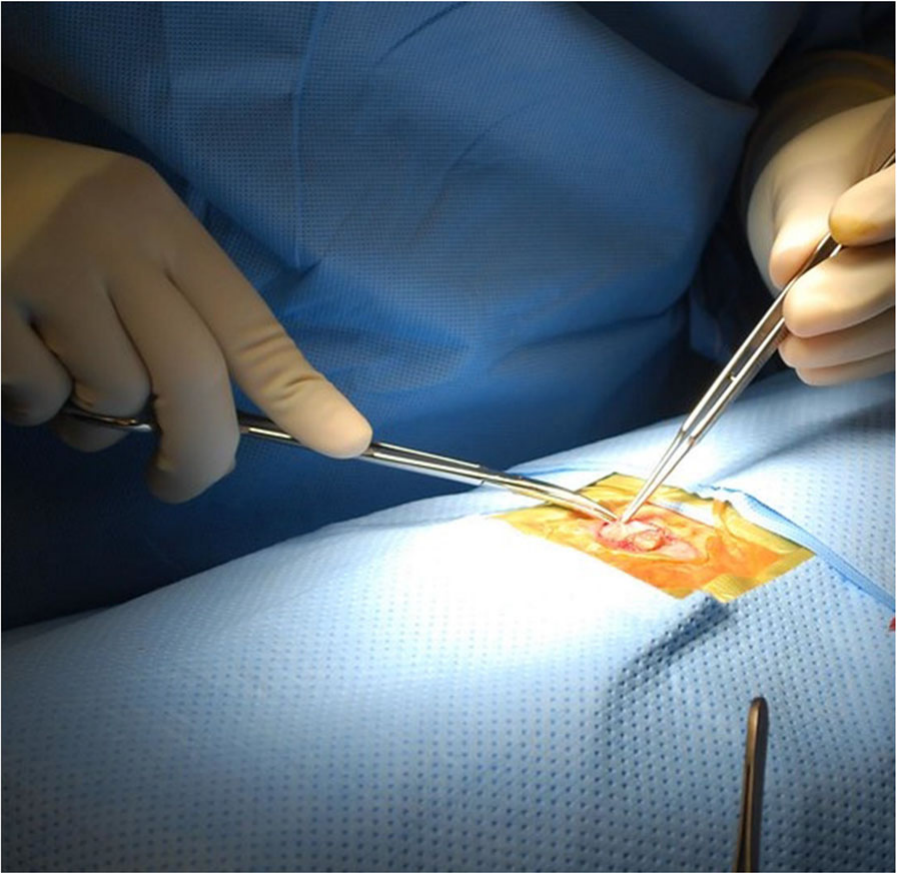
Surgical excision of umbilical endometriosis

All patients had surgical excision of the lesion (Fig. 2) performed by a consultant gynecologist with a 1-cm safety margin up to the rectus fascia with closure of the umbilical defect. Three patients had additional surgery. One had laparoscopy and hysteroscopy due to dysmenorrhea, which were normal. Another patient with subfertility, dysmenorrhea, and heavy menstrual bleeding had laparoscopy, where pelvic endometriosis was found with lesions on the uterosacral ligaments, right ovarian fossa, and posterior uterine wall, which were ablated. This patient also had a total laparoscopic hysterectomy. The last patient had laparoscopy with ablation and excision of superficial endometriosis deposits on the anterior and posterior cul-de-sac.

Histopathological examination was undertaken in all the specimens excised, and endometriosis was confirmed by identification of endometrial glands and/or stroma and recent hemorrhage in the dermis with no malignancy. All patients had uneventful postoperative recoveries with no recurrence in follow-up.

## Discussion and conclusions

The average age of the five patients was 40 years, whereas that reported in the literature is 37.7 ± 0.98 years [4]. This indicates that endometriosis is an estrogen-dependent condition affecting premenopausal women of reproductive age [7]. The mean duration of the symptoms was 21.4 years, which is consistent with that in the literature of 17.8 ± 3.9 months [4]. Umbilical endometriosis can be primary if it occurs spontaneously or secondary following previous surgery, especially laparoscopic procedures with umbilical port entry. None of the five patients had a prior laparoscopic surgery; however, one had two prior cesarean sections. Secondary umbilical endometriosis can occur following cesarean sections in 1% of cases [8].

The diagnosis is often made on the basis of clinical presentation, which was consistent with that reported in the literature. Most patients present with umbilical swelling with cyclical pain and bleeding. According to Victory *et al.* [4], umbilical swelling was present in almost 90% of cases with less than 50% having bleeding and about 80% having pain. Pain is caused by tissue inflammation, distention, and cyclical changes. The mean size of the lesion is about 2.29 cm, with color changes ranging from brown to blue, purple, black, and normal in decreasing order [4]. In our study, however, the color of the lesions was not reported. Discoloration occurs as a result of bleeding into the lesion with hemosiderin deposition, which can be seen at histopathological examination.

Although preliminary diagnosis is made on the basis of history and physical examination, imaging may aid in preoperative evaluation. Ultrasound can be used to assess the nodule size and involvement of surrounding tissues and to evaluate other pelvic pathology, hence aiding the planning of surgical management [9]. One of the patients had a pelvic ultrasound. In this patient, the ultrasound features of umbilical endometriosis, which include isoechoic region with hyperechoic foci with or without abundant blood supply on Doppler [9], were not seen. However, other pelvic pathology, including intramural fibroids and ovarian cyst, were discovered. MRI can also be used as a method of preoperative evaluation in suspected endometriosis. It aids in evaluating pelvic endometriosis as well as to rule out other sinister differentials, including malignancy, Sister Mary Joseph nodule, and granuloma, among others. MRI features of an umbilical endometriosis includes a homogeneous hypointense lesion on T1-weighted sequence with low signals on T2 weighting [10, 11]. One of the patients in this study had an MRI scan with features of umbilical endometriosis.

Up to 25% of umbilical endometriosis occurs with concurrent pelvic endometriosis. Two patients had coexisting pelvic endometriosis treated at laparoscopy. Subfertility is a common condition among patients with endometriosis, occurring in up to 50% of women with endometriosis [1]. This phenomenon was reported in one patient in this study who was seen in follow-up for subfertility with bilateral tubal blockage.

The umbilicus is a physiological scar that is a preferred site for umbilical endometriosis, as described by Yu *et al.* [11] Lymphatic and hematogenous spread to the umbilicus and direct extension of endometrial cells through round ligaments or omphalomesenteric remnants are possible theories to explain the etiology of umbilical endometriosis [6, 12].

Surgical management is the treatment of choice [5, 12, 13]. Hormone therapy can be used preoperatively for relief of symptoms, but it is not curative. It can also be used to reduce the size of large lesions prior to surgery. However, it is associated with side effects such as amenorrhea [13]. Surgical excision was the treatment administered to the patients in this study. These lesions have a low risk of malignancy and recurrence [4, 14]. Diagnosis is confirmed by histopathological examination.

Umbilical endometriosis is a rare entity, especially when it occurs spontaneously. The clinical presentation of umbilical swelling, cyclical pain, and sometimes bleeding from the lesion are highly suggestive of this condition. The treatment of choice is surgical excision, and diagnosis is confirmed by histopathological examination.

## Data Availability

Clinical data and complementary examinations are available from the corresponding author on reasonable request.

## Abbreviation

MRI: Magnetic resonance imaging
